# Real-World Comparison of Stroke Practitioner-Led and Neurohospitalist-Led Acute Ischemic Stroke Workflows †

**DOI:** 10.3390/healthcare14131989

**Published:** 2026-07-03

**Authors:** Hatice Yelda Yıldız, Yavuz Bekmezci, Ali Sağlık, Tarık Ocak, Umut Esen, Gamze Keskin, Gülşah Kayhan, Neslihan Oral, Birol Balkan, Serpil Çıracı, Yakup Krespi

**Affiliations:** 1 Department of Neurology, Istinye University Hospital, Istanbul 34396, Türkiye; y.yavuzbekmezci@gmail.com (Y.B.); gulsah.yilmaz1@isu.edu.tr (G.K.); yakup.krespi@istinye.edu.tr (Y.K.); 2 Department of Emergency Medicine, Istinye University Hospital, Istanbul 34396, Türkiye; ali.saglik@isu.edu.tr (A.S.); tarik.ocak@isu.edu.tr (T.O.); 3 Department of Family Medicine, Istinye University Hospital, Istanbul 34396, Türkiye; umut.esen@isu.edu.tr (U.E.); gamze.keskin1@isu.edu.tr (G.K.); 4 Quality Management Department, Istinye University Hospital, Istanbul 34396, Türkiye; neslihan.oral1@isu.edu.tr; 5 Department of Radiology, Istinye University Hospital, Istanbul 34396, Türkiye; 1olbalkan@gmail.com; 6 Department of Pharmacy Services, Istinye University Hospital, Istanbul 34396, Türkiye; serpil.ciraci@isu.edu.tr

**Keywords:** acute ischemic stroke, stroke workflow, door-to-needle time, multidisciplinary care, stroke systems, reperfusion therapy

## Abstract

**Highlights:**

**What are the main findings?**

- Major safety and functional outcomes were comparable between workflow periods.
- The stroke practitioner-led period was independently associated with lower ICU transfer rates.

**What are the implications of the main findings?**

- Structured multidisciplinary workflows may support efficient stroke care despite limited neurologist availability.
- Practitioner-led coordination may optimize resource utilization without compromising clinical outcomes.

**Abstract:**

**Background/Objectives**: Acute ischemic stroke (AIS) care depends on rapid, coordinated workflows. This study compared two real-world in-hospital stroke models—a neurohospitalist-led model and a stroke practitioner-led multidisciplinary model—in terms of time metrics, radiological outcomes, and 3-month clinical outcomes in patients undergoing reperfusion therapy. **Methods**: This retrospective, single-center cohort study evaluated patients across two sequential workflow periods. In the practitioner-led model, trained non-neurologist clinicians coordinated care with a stroke nurse under neurologist supervision. Time metrics included door-to-needle time (DNT) and door-to-puncture time (DPT). Clinical outcomes included intensive care unit (ICU) transfer and 3-month functional outcomes assessed by the modified Rankin Scale (mRS). Multivariable logistic regression analyses were performed to explore variables associated with achievement of DNT < 60 min and ICU transfer. **Results**: A total of 573 patients were included (284 neurohospitalist-led, 289 practitioner-led). Baseline NIHSS scores were similar between groups. Among patients receiving intravenous thrombolysis, the proportion achieving DNT < 60 min did not differ significantly between periods (77.9% vs. 72.5%, *p* = 0.124), while mean DNT and DPT were comparable. Early radiological outcomes at 24 h were similar between groups. ICU transfer rates were significantly lower in the practitioner-led period (17.6% vs. 28.2%, *p* = 0.002). In multivariable analyses, the stroke practitioner-led period was not independently associated with achieving DNT < 60 min among thrombolysed patients, but remained independently associated with a lower likelihood of ICU transfer. Three-month mRS outcomes did not differ significantly. **Conclusions**: A structured, practitioner-led multidisciplinary workflow was associated with lower ICU transfer rates, while no statistically significant differences were detected in DNT target achievement among thrombolysed patients, safety outcomes, or functional outcomes compared with the neurohospitalist-led period. The observed associations between workflow organization and ICU utilization highlight the potential importance of system-level factors in AIS care delivery.

## 1. Introduction

Acute ischemic stroke (AIS) remains a major cause of mortality and disability worldwide and continues to impose a substantial clinical and organizational burden on health systems. In the Turkish context, stroke is also a major vascular health problem, and recent work from Türkiye has further emphasized the importance of structured stroke-related assessment and early complication management in local clinical practice [1]. Contemporary AIS management is fundamentally based on rapid recognition, urgent imaging, timely reperfusion, and coordinated in-hospital care, as reflected in current guideline recommendations that support organized stroke systems of care across prehospital and hospital settings [2].

The therapeutic benefit of reperfusion therapy in AIS is highly time-dependent. Pooled analyses and meta-analyses of alteplase trials have consistently shown that earlier intravenous thrombolysis (IVT) is associated with greater functional benefit, whereas treatment delays diminish efficacy and may worsen overall outcomes [3,4,5]. Likewise, advances in endovascular thrombectomy (EVT) have expanded treatment opportunities for selected patients with large vessel occlusion (LVO), including those treated in extended windows when supported by advanced imaging-based selection, reinforcing the need for streamlined and responsive stroke workflows throughout the acute care pathway [6].

Because treatment benefit is tightly linked to speed, optimization of stroke workflow has become a central objective of modern stroke centers. Large quality-improvement initiatives such as Target: Stroke have demonstrated that reducing door-to-needle time (DNT) at scale is feasible and is associated with better clinical outcomes, including lower in-hospital mortality and reduced symptomatic intracranial hemorrhage [7,8]. Subsequent studies have further shown that specific operational strategies—including direct transport to computed tomography, rapid registration, scanner-based alteplase administration, pre-notification systems, and structured team activation—can substantially shorten treatment times in routine clinical practice [9,10,11]. Recent studies continue to demonstrate that workflow optimization remains a major determinant of treatment efficiency in AIS, with increasing emphasis on identifying specific workflow bottlenecks and implementing targeted organizational interventions to improve door-to-treatment performance [12,13].

Importantly, workflow improvement in stroke care is not limited to physician-led decision-making alone but depends on a multidisciplinary and system-oriented approach. Lean redesign models, collaborative hospital interventions, and structured team-based protocols have all been associated with shorter time-to-thrombolysis and, in some settings, improved functional outcomes [14,15,16,17]. More recent evidence also suggests that nursing-driven and protocolized stroke care models can improve selected time-sensitive workflow metrics, even if gains in thrombolysis timing are not universal, underscoring the importance of clearly defined responsibilities and standardized coordination across the entire stroke pathway [18].

Despite these advances, the most effective real-world configuration of acute stroke teams may vary across institutions depending on patient volume, staffing constraints, available expertise, and local organizational structure, particularly in developing stroke systems. In particular, there remains limited real-world evidence on how evolving organizational models—from traditional neurohospitalist-led systems to structured multidisciplinary models led by stroke practitioners (hospitalist general practitioners) in coordination with specialized stroke nurses under neurologist supervision—affect time metrics and clinical outcomes within the same institutional setting. Therefore, this study aimed to compare two sequential real-world stroke workflow periods at a single comprehensive stroke center—a neurohospitalist-led period and a stroke practitioner-led period—with respect to time metrics and 3-month clinical outcomes in patients with AIS.

## 2. Materials and Methods

### 2.1. Study Design

This retrospective, single-center cohort study was conducted at the Istinye University Liv Hospital Bahçeşehir Comprehensive Stroke Center between January 2020 and December 2024. Although the analysis was retrospective, all stroke cases were prospectively, systematically, and consecutively recorded within the continuously maintained Istinye Stroke Registry, which operates as a high-quality, standardized data capture system integrated into routine clinical practice. This structured and consistently audited registry enabled precise delineation of workflow periods based on the natural evolution of the stroke center rather than predefined interventional phases. The workflow periods were defined according to sequential organizational models implemented during routine clinical practice. Each period reflects a distinct staffing and workflow configuration used for the management of acute ischemic stroke within the same comprehensive stroke center.

### 2.2. Participants

**Figure 1:**
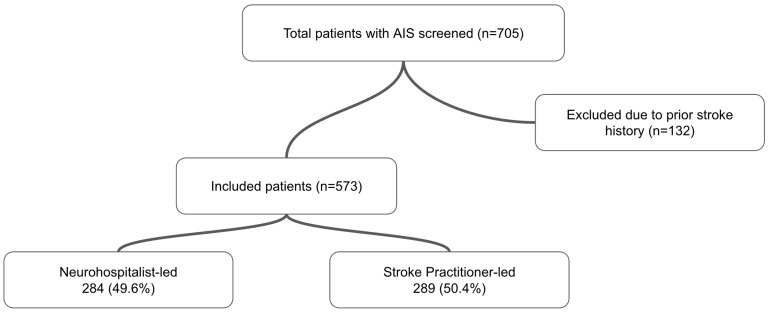
Study flow diagram. The flowchart illustrates the screening of patients with acute ischemic stroke (AIS), exclusion of patients with prior stroke history, and final cohort selection. A total of 705 patients were screened, of whom 132 were excluded due to prior stroke history, resulting in a final cohort of 573 patients. These patients were subsequently allocated to the neurohospitalist-led period (*n* = 284) and the stroke practitioner-led period (*n* = 289).

A total of 705 patients were screened, of whom 132 were excluded due to prior stroke history, resulting in a final cohort of 573 patients (Figure 1). Eligible participants were adults aged ≥18 years presenting with AIS and undergoing reperfusion therapy, including IVT, EVT, or both. Patients were consecutively included from the institutional stroke registry. Patients with a prior history of stroke were excluded to reduce the influence of pre-existing neurological disability and prior stroke-related functional impairment on outcome assessment. Because prior stroke may independently affect baseline functional status, ICU utilization, recovery trajectories, and 3-month mRS outcomes, exclusion of these patients was intended to improve cohort homogeneity and allow a more reliable evaluation of workflow-related associations.

### 2.3. Istinye Stroke Registry

All AIS patients managed by the stroke team were entered prospectively and consecutively into the Istinye Stroke Registry. The registry includes demographic, clinical, radiologic, time-metric, treatment, and functional outcome data, enabling retrospective data analyses.

### 2.4. Rationale for Defining Stroke Workflow Periods

The stroke workflow at the Istinye University Comprehensive Stroke Center underwent progressive organizational modifications during the study period in response to operational needs and increasing patient volume. Accordingly, the workflow periods analyzed in this study represent two sequential organizational models of acute stroke care. These periods were defined to compare differences in team structure, workflow coordination, and treatment delivery within the same institutional setting.

### 2.5. Stroke Workflow Periods

**Figure 2:**
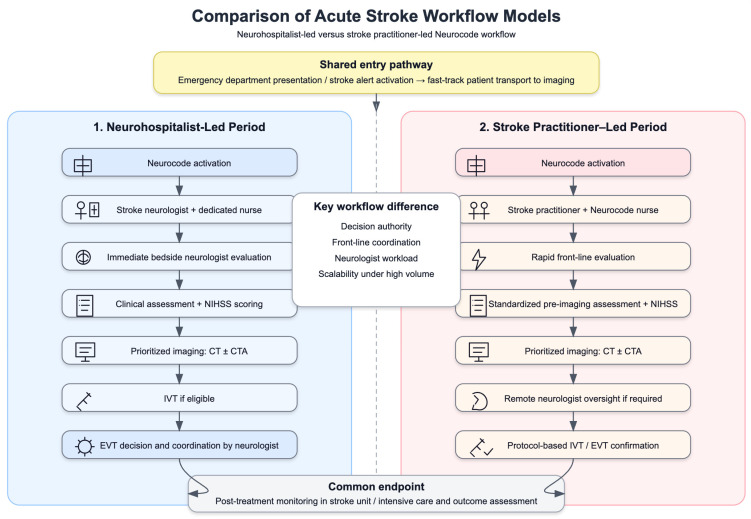
Schematic comparison of two sequential acute stroke workflow models. The Neurohospitalist-led model (**left**) is based on real-time bedside evaluation and decision-making by an on-site stroke neurologist. The stroke practitioner-led model (**right**) incorporates trained non-neurologist clinicians for front-line assessment and workflow coordination, with remote neurologist oversight. Both models share standardized imaging pathways and converge on post-treatment monitoring and outcome assessment. Abbreviations: AIS, acute ischemic stroke; ED, emergency department; CT, computed tomography; CTA, computed tomography angiography; IVT, intravenous thrombolysis; EVT, endovascular treatment; NIHSS, National Institutes of Health Stroke Scale.

Two sequential workflow periods were defined based on the natural evolution of the institutional stroke care system (Figure 2).

1. Neurohospitalist-Led Period

During this period, a Neurocode system composed of a stroke neurologist and a dedicated stroke nurse performed real-time evaluation, diagnosis, treatment decision-making, and operational coordination of all AIS cases. Early workflow support included structured hospital-wide Neurocode alerts and a fast-track patient transport pathway enabling prioritized transfer to imaging.

2. Stroke Practitioner–Led Period

In this period, an advanced team-based workflow model was implemented in response to increasing patient volume and resource constraints. Specially trained non-neurologist clinicians were incorporated into the Neurocode team as stroke practitioners, enabling rapid front-line evaluation and workflow coordination. A stroke practitioner and a dedicated Neurocode nurse jointly managed patient flow, while a remotely available supervising stroke neurologist provided oversight and confirmed treatment decisions when required. Pre-imaging and imaging assessments were standardized across both periods.

Stroke practitioners were physicians from emergency medicine and family medicine backgrounds who underwent a structured 6-month institutional stroke training program consisting of neurological examination training, NIHSS certification, acute stroke recognition, stroke workflow management, neuroimaging review, and reperfusion eligibility assessment. Following completion of training, stroke practitioners participated in acute stroke care under the continuous supervision of a vascular neurologist. Their responsibilities included front-line patient assessment, activation and coordination of the Neurocode pathway, collection of relevant clinical information, and facilitation of imaging and treatment readiness. Final treatment decisions regarding intravenous thrombolysis and endovascular therapy remained under neurologist supervision. Accordingly, the practitioner-led workflow represented a model of front-line practitioner coordination with continuous neurologist availability and specialist backup rather than an independent practitioner-only stroke service. Neurologist consultation was available at all times and was required whenever reperfusion eligibility, imaging interpretation, or treatment strategy required specialist confirmation.

### 2.6. Data Collection and Variables

Extracted variables included demographics, clinical characteristics (National Institutes of Health Stroke Scale [NIHSS], vascular risk factors), and imaging data (LVO presence). Time metrics included last known well time, wake-up stroke status, DNT, and DPT. Radiologic outcomes included hemorrhagic transformation, parenchymal hematoma, and remote hemorrhage, with symptomatic intracranial hemorrhage defined according to the Safe Implementation of Treatments in Stroke–Monitoring Study (SITS-MOST) criteria [19].

The primary workflow outcome was the proportion of patients achieving DNT < 60 min among patients receiving intravenous thrombolysis. Secondary outcomes included DPT, Intensive care unit (ICU) transfer, radiological outcomes, and 3-month functional outcomes (mRS).

For secondary analyses, the mRS at 3 months was further categorized into three clinically meaningful groups: no disability (mRS 0–2), disability present (mRS 3–5), and death (mRS 6). This categorization was used to facilitate comparison of functional outcomes between workflow models.

### 2.7. Potential Sources of Bias

Potential sources of bias included differences in baseline characteristics between workflow periods, inclusion of patients during daytime hours when neurologists were available in both periods, and possible contamination due to neurologist consultation in the stroke practitioner-led model.

### 2.8. Statistical Analysis

Statistical analyses were performed using Wistats v3.0 (WisdomEra Corp., Istanbul, Turkey), a Python-based statistical analysis platform. Wistats was used as the analytical environment for implementing the statistical procedures described in this study. (https://wistats.wisdomera.io, accessed on 18 May 2026). Data distribution was evaluated using skewness and kurtosis statistics, while the Shapiro–Wilk test was applied to assess normality. Selection of statistical tests for comparative analyses was based on data distribution characteristics. Accordingly, non-parametric methods were used for variables that did not satisfy assumptions of normal distribution.

Comparative analyses were conducted between the neurohospitalist-led and stroke practitioner-led periods across predefined variable groups, including demographic variables (age, sex), clinical characteristics (NIHSS, vascular risk factors), imaging findings, presence and subtype of LVO, workflow time metrics (last known well time, DNT, DPT), and radiological outcomes (hemorrhagic transformation, infarct expansion, edema, and recanalization).

Comparisons between categorical variables were conducted using Chi-square or Fisher’s Exact tests. Analyses involving numerical variables were performed using the Independent Samples *t*-test or Mann–Whitney U test for two-group comparisons, and One-way ANOVA or Kruskal–Wallis tests where appropriate. Time metrics were analyzed both as continuous and categorical variables. Time metrics were additionally summarized using median (IQR) because of non-normal distributions.

Multivariable logistic regression analyses were performed for two key workflow-related outcomes: achievement of DNT < 60 min and ICU transfer. Candidate variables were selected a priori based on clinical relevance and their potential to confound the association between workflow period and study outcomes. The DNT < 60 min model included stroke workflow period, age, baseline NIHSS score, treatment type, and LVO status. The ICU transfer model included stroke workflow period, age, baseline NIHSS score, treatment type modeled using indicator variables, and LVO status. DNT category was not included in the final ICU transfer model because DNT is inherently undefined for EVT-only patients. Results are presented as odds ratios (ORs) with 95% confidence intervals (CIs).

All statistical tests were two-tailed, and *p* values < 0.05 were considered statistically significant. No formal sample size calculation was performed, as all eligible patients within the study period were included.

### 2.9. Handling of Missing Data

Missing data were not imputed. All analyses were conducted using an available-case (complete-case) approach, whereby each variable was analyzed based on non-missing observations. Accordingly, the number of observations may vary across analyses. To improve transparency regarding the extent and distribution of missing data, the number of available and missing observations for key variables was summarized separately for each workflow period and is presented in Supplementary Table S1.

## 3. Results

### 3.1. Baseline Characteristics of the Study Population

A total of 573 patients with AIS were included in the study, of whom 284 (49.6%) were managed during the neurohospitalist-led period and 289 (50.4%) during the stroke practitioner-led period (Table 1). The overall mean age was 68.79 ± 13.77 years, and 45.7% of the cohort were female. The mean baseline NIHSS score was 11.91 ± 6.45, indicating a moderate stroke severity profile.

The distribution of cases across years reflected a temporal transition in workflow organization, with earlier years dominated by the neurohospitalist-led model and later years by the stroke practitioner-led model. Additional baseline characteristics, including admission day, duty period, treatment type, and time metrics, are summarized in Table 1.

### 3.2. Comparison of Clinical Characteristics Between Workflow Periods

Baseline stroke severity, as assessed by NIHSS, was comparable between the two periods (12.09 ± 6.35 vs. 11.72 ± 6.55, *p* = 0.486). However, patients managed during the stroke practitioner-led period were significantly older than those in the neurohospitalist-led period (70.73 ± 13.06 vs. 66.82 ± 14.20 years, *p* = 0.002) (Table 2).

The last known well time was significantly shorter in the stroke practitioner-led period (217.71 ± 250.07 vs. 292.79 ± 236.22 min, *p* < 0.001). In addition, wake-up strokes were more frequent in the stroke practitioner-led period (5.1% vs. 1.5%, *p* = 0.029).

**Figure 3:**
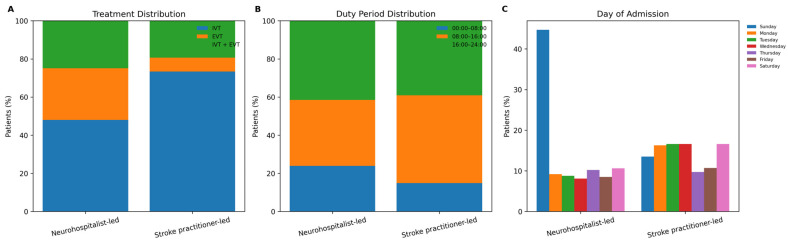
Treatment patterns and workflow context by period. (**A**) Distribution of treatment modalities (intravenous thrombolysis [IVT], endovascular thrombectomy [EVT], and combined IVT + EVT). (**B**) Distribution of duty periods (00:00–08:00, 08:00–16:00, 16:00–24:00). (**C**) Distribution of admission days across the week. Treatment strategies and workflow-related contextual variables are presented descriptively to illustrate differences between workflow periods.

Treatment strategies differed significantly between the two periods (*p* < 0.001), with a higher proportion of IVT in the stroke practitioner-led period (73.4% vs. 48.0%), whereas EVT was more commonly performed during the neurohospitalist-led period (27.1% vs. 7.3%). Combined IVT + EVT treatment rates were relatively similar (Figure 3A).

Significant differences were also observed in admission timing variables. The distribution of admission days varied between periods (*p* < 0.001), as did duty periods (*p* = 0.005), suggesting potential differences in workflow dynamics, referral patterns, staffing characteristics, or operational factors between study periods (Figure 3B,C).

Despite these differences, LVO rates were comparable between groups (76.4% vs. 71.5%, *p* = 0.108).

### 3.3. Time Metrics and Workflow Performance

**Figure 4:**
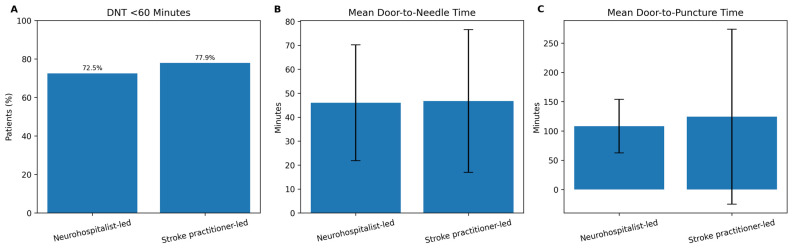
Workflow performance between neurohospitalist-led and stroke practitioner-led periods. (**A**) Proportion of patients achieving door-to-needle time (DNT) < 60 min. (**B**) Mean DNT (minutes) with standard deviation. (**C**) Mean door-to-puncture time (DPT) (minutes) with standard deviation. The figure provides a descriptive comparison of workflow performance metrics between the two study periods.

Key time metrics were largely similar between the two workflow models. Among patients receiving intravenous thrombolysis, DNT did not differ significantly between periods (46.01 ± 24.18 vs. 46.76 ± 29.76 min, *p* = 0.952). When analyzed categorically in the thrombolysis population, the proportion of patients achieving DNT < 60 min was numerically higher in the stroke practitioner-led period but did not reach statistical significance (77.9% vs. 72.5%, *p* = 0.124) (Figure 4).

DPT was also comparable between groups (108.22 ± 45.71 vs. 124.36 ± 149.46 min, *p* = 0.704).

### 3.4. Early Radiological Outcomes

Early radiological outcomes at 24 h were similar between the two workflow periods (Table 3). There were no significant differences in infarct expansion (*p* = 0.451), hemorrhagic transformation patterns (*p* = 0.982), remote infarction (*p* = 0.746), or brain edema (*p* = 0.330).

Recanalization rates showed no statistically significant difference between groups (*p* = 0.087), although numerically higher recanalization was observed in the neurohospitalist-led period.

### 3.5. Clinical Outcomes

**Figure 5:**
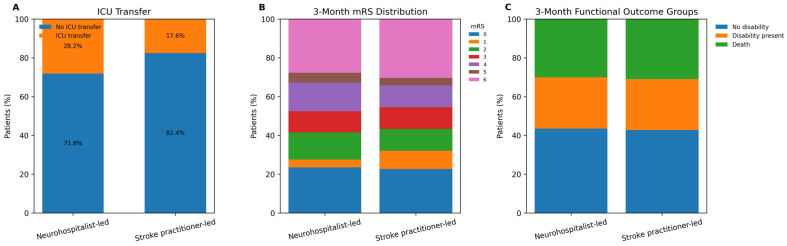
Early and late clinical outcomes between neurohospitalist-led and stroke practitioner-led periods. (**A**) Intensive care unit (ICU) transfer rates. (**B**) Distribution of 3-month modified Rankin Scale (mRS) scores (0–6). (**C**) Grouped 3-month functional outcomes (no disability, disability present, death). The figure presents descriptive comparisons of clinical outcomes observed across the two workflow periods.

Clinical outcomes were largely comparable between the two models. Three-month functional outcomes, as measured by the mRS, did not differ significantly between groups (*p* = 0.208) (Table 3 and Figure 5). Similarly, when categorized into disability groups, no significant differences were observed (*p* = 0.490).

However, ICU transfer rates were significantly lower in the stroke practitioner-led period compared to the neurohospitalist-led period (17.6% vs. 28.2%, *p* = 0.002). This finding should be interpreted cautiously, as differences in treatment distribution—particularly the substantially higher proportion of EVT-treated patients during the neurohospitalist-led period—may have influenced ICU utilization independently of workflow organization.

### 3.6. Multivariable Analyses of Workflow Performance and ICU Transfer

To further explore factors associated with workflow performance and ICU utilization, multivariable logistic regression analyses were performed (Table 4 and Table 5).

In the model evaluating achievement of DNT < 60 min among patients receiving intravenous thrombolysis, the stroke practitioner-led workflow period was not independently associated with achieving the target DNT compared with the neurohospitalist-led period (OR 1.44, 95% CI 0.91–2.30, *p* = 0.122). LVO was also not independently associated with achievement of DNT < 60 min (OR 0.69, 95% CI 0.40–1.18, *p* = 0.179). Age, baseline NIHSS score, and treatment type were not independently associated with achievement of the DNT target.

In the model evaluating ICU transfer, the stroke practitioner-led workflow period was independently associated with a lower likelihood of ICU transfer compared with the neurohospitalist-led period (OR 0.57, 95% CI 0.36–0.90, *p* = 0.015). Higher baseline NIHSS scores (OR 1.11, 95% CI 1.07–1.15, *p* < 0.001) and older age (OR 1.02, 95% CI 1.01–1.04, *p* = 0.006) were independently associated with an increased likelihood of ICU transfer. Treatment type was modeled using indicator variables with IVT as the reference category. EVT only was independently associated with a higher likelihood of ICU transfer compared with IVT (OR 1.89, 95% CI 1.04–3.44, *p* = 0.037), whereas IVT + EVT was not significantly associated with ICU transfer (OR 1.16, 95% CI 0.67–2.03, *p* = 0.593). LVO was not independently associated with ICU transfer.

## 4. Discussion

In this study, we evaluated the impact of two sequential real-world stroke workflow models—a neurohospitalist-led model and a stroke practitioner-led multidisciplinary model—on time metrics, safety, and clinical outcomes in patients with AIS. The most important findings can be summarized as follows: (i) although mean DNT and DPT were similar between groups, (ii) among patients receiving intravenous thrombolysis, the proportion achieving DNT < 60 min was numerically higher in the stroke practitioner-led period but did not reach statistical significance, (iii) ICU transfer rates were significantly lower in the stroke practitioner-led period, and (iv) 3-month functional outcomes (mRS) were comparable between groups. Taken together, these findings suggest that the stroke practitioner-led workflow period was associated with lower ICU transfer rates, while DNT target achievement among thrombolysed patients and safety and functional outcomes did not differ significantly between workflow periods. However, the study was not designed or powered to establish equivalence between workflow models.

Time-dependent benefit remains the cornerstone of AIS care, as consistently demonstrated in pooled analyses and meta-analyses showing that earlier IVT leads to better outcomes and reduced mortality [3,4]. In our study, among patients receiving intravenous thrombolysis, mean DNT did not differ significantly between workflow periods, and the proportion treated within the guideline-recommended 60 min window was numerically higher in the stroke practitioner-led period but did not reach statistical significance (77.9% vs. 72.5%). This finding aligns with large-scale quality improvement initiatives such as Target: Stroke, where increasing the proportion of patients treated within 60 min has been associated with improved outcomes, including reduced mortality [8,20]. Recent evaluations of the Target: Stroke program continue to emphasize achievement of DNT benchmarks as a key quality indicator in contemporary stroke systems [21]. Although DNT < 60 achievement was numerically higher in the practitioner-led period, this difference did not remain significant after multivariable adjustment. In multivariable analyses restricted to patients receiving intravenous thrombolysis, the stroke practitioner-led workflow period was not independently associated with achievement of DNT < 60 min, and LVO was also not independently associated with the DNT target. Age, NIHSS score, and treatment type were not independently associated with achievement of DNT < 60 min. These findings suggest that, after restricting the analysis to patients with an applicable DNT measure and adjusting for measured case-mix variables, the workflow period was not independently associated with DNT target attainment. However, the coexistence of similar mean DNT values and different DNT < 60 min rates suggests that the underlying distributions may have differed between workflow periods.

The numerically higher DNT target achievement in the stroke practitioner-led period, although not statistically significant, is consistent with prior studies emphasizing the importance of structured protocols and multidisciplinary coordination. Strategies such as direct transfer to imaging, pre-notification systems, and standardized stroke code activation have been shown to significantly reduce treatment delays [9,10]. Similarly, meta-analytic data indicate that organizational interventions and comprehensive stroke pathways can substantially increase IVT utilization and improve adherence to time targets [22]. The workflow model implemented in our center incorporates many of these elements, including rapid activation, parallel processing, and clearly defined team roles, which may have been associated with the observed differences in workflow metrics.

Despite the numerically higher DNT target achievement, 3-month functional outcomes were similar between groups. It should also be noted that overall mortality at 3 months was relatively high in the study cohort. This likely reflects the inclusion of patients requiring reperfusion therapy and the high prevalence of LVO, indicating a clinically high-risk population despite a moderate mean NIHSS score. This finding may reflect the influence of several important differences between workflow periods, including older age in the stroke practitioner-led group, differences in last-known-well time, wake-up stroke frequency, treatment distribution, admission timing, duty periods, and other workflow-related characteristics that may have influenced both treatment processes and outcomes. Previous studies have shown that while workflow optimization can reduce early complications and mortality, long-term functional outcomes are also shaped by factors such as infarct size, collateral circulation, and post-acute care processes [23]. Therefore, the absence of a difference in long-term outcomes suggests that the lower ICU transfer rate should be interpreted cautiously and may reflect differences in treatment distribution, monitoring pathways, resource utilization, institutional practice, or patient characteristics rather than a direct reduction in clinical deterioration. Multivariable analysis further demonstrated that the stroke practitioner-led workflow period remained independently associated with a lower likelihood of ICU transfer after adjustment for age, NIHSS score, treatment type, and LVO status. Higher NIHSS scores, older age, and EVT-only treatment were independently associated with ICU transfer, whereas IVT + EVT and LVO were not independently associated with ICU transfer. Nevertheless, because detailed indications for ICU admission were not available, this association should be interpreted cautiously and should not be considered evidence of a causal reduction in clinical deterioration.

The integration of a multidisciplinary stroke team led by trained stroke practitioners represents an important organizational adaptation, particularly in healthcare systems where neurologist availability is limited. Expanding team roles and distributing responsibilities may enhance system resilience, scalability, and responsiveness to increasing patient volumes. Prior studies have demonstrated that protocol-driven and team-based stroke care models can improve efficiency even in resource-constrained environments [24]. In this context, our findings suggest that such models may provide a viable and sustainable alternative to exclusively neurologist-led systems.

Beyond traditional workflow metrics, the effectiveness of multidisciplinary stroke care models may also be influenced by how patient care complexity is distributed across the healthcare team. Recent evidence has demonstrated that nursing complexity and nursing intensity vary substantially across ischemic stroke, hemorrhagic stroke, and transient ischemic attack populations and may not be fully explained by medical severity alone. This perspective suggests that organizational performance may depend not only on treatment speed but also on the ability of multidisciplinary teams to absorb and manage varying levels of care demand. Future studies evaluating stroke workflows should consider incorporating standardized nursing complexity and nursing intensity indicators to better characterize the mechanisms through which different organizational models influence patient care and resource utilization [25].

Several limitations should be acknowledged. First, this was a single-center, retrospective analysis, which may limit generalizability. Second, although patients were prospectively recorded, unmeasured confounders and temporal changes in clinical practice may have influenced outcomes. The before–after design represents an important limitation of the study. Because the two workflow models were implemented during consecutive organizational periods rather than through random allocation, the observed differences may have been influenced not only by the workflow structure itself but also by secular trends and ongoing institutional development. Factors such as increasing team experience, continuous staff training, progressive refinement of operational processes, technological improvements, and evolving stroke care practices may have contributed to the observed improvements over time. Therefore, the findings should be interpreted as reflecting the performance of two real-world organizational models within a continuously developing stroke system rather than establishing a direct causal relationship between workflow structure and clinical outcomes. Although multivariable regression analyses were performed for key workflow-related outcomes, residual confounding cannot be excluded because not all potential differences between periods could be measured or adjusted for. Consequently, the observed associations should be interpreted cautiously. Future multicenter studies using contemporaneous comparison groups or more robust study designs may help further distinguish workflow-related effects from temporal organizational changes. Consequently, some of the observed differences between periods, particularly those related to ICU utilization, may partly reflect broader healthcare system changes rather than the workflow model alone. Third, the study design does not allow for causal inference regarding specific components of the workflow, and the relative contribution of each intervention within the workflow model could not be isolated. Furthermore, multiple comparisons were performed across demographic, workflow, radiological, and clinical outcome variables without formal adjustment for multiplicity. Therefore, statistically significant findings, particularly among secondary outcomes, should be interpreted cautiously and regarded as hypothesis-generating. Fourth, a potential source of bias is that patients admitted during daytime hours—when a neurologist was actively involved in both workflow periods—were included in the analysis, which may have attenuated differences between groups. In addition, several clinically relevant differences between workflow periods, including age, treatment distribution, last-known-well time, wake-up stroke frequency, admission timing, duty periods, and potential differences in referral patterns, may have contributed to outcome variability and represent important sources of residual confounding. Furthermore, missing data were not uniformly distributed across variables and workflow periods. Because analyses were conducted using an available-case approach without imputation, differential or potentially non-random missingness may have influenced some comparisons and represents an additional source of bias. Furthermore, during the stroke practitioner-led period, consultation with a neurologist was available when necessary, and treatment decisions could be made collaboratively; this may have introduced a contamination effect, further reducing observable differences between workflow models. Finally, long-term outcomes beyond 3 months, healthcare utilization metrics such as hospital length of stay, ICU stay duration, and readmission rates, as well as detailed process-level metrics (e.g., prehospital delays), were not evaluated. Inclusion of these measures in future studies may provide a more comprehensive assessment of the organizational and resource-related impact of different stroke workflow models. Future studies stratifying patients according to duty periods, adjusting for comorbidity burden, minimizing cross-model interactions, and incorporating propensity-based approaches and ordinal outcome analyses are warranted to further clarify workflow-related associations.

In addition, part of the study period overlapped with the COVID-19 pandemic, which may have influenced emergency department operations, ICU admission practices, resource allocation, referral patterns, and treatment workflows. Consequently, some of the observed differences between periods, particularly those related to ICU utilization, may partly reflect broader healthcare system changes rather than the workflow model alone. Because the workflow periods were defined chronologically, pandemic-related effects represent an additional source of temporal confounding that cannot be fully separated from the organizational changes evaluated in this study.

Future studies employing propensity-based approaches and ordinal outcome analyses may further clarify the associations observed between workflow organization and clinical outcomes. Future research should focus on multicenter validation of structured multidisciplinary stroke workflow models, with particular emphasis on identifying the most impactful components. Integration of advanced decision-support tools, automated alert systems, and real-time performance monitoring may further enhance workflow efficiency and consistency.

## 5. Conclusions

A structured, multidisciplinary workflow led by stroke practitioners was associated with lower ICU transfer rates, while no statistically significant differences were detected in DNT target achievement among thrombolysed patients, safety outcomes, or functional outcomes compared with the neurohospitalist-led period. Numerically higher DNT target achievement among thrombolysed patients and lower ICU transfer rates were observed during the stroke practitioner-led period; however, these findings should be interpreted cautiously given the observational design and residual confounding.

While no statistically significant differences were detected in long-term functional outcomes (3-month mRS) between groups, these findings suggest a potential association between system-level organization, multidisciplinary coordination, and selected workflow outcomes in AIS care.

These findings generate hypotheses regarding the potential role of practitioner-led multidisciplinary workflows in supporting stroke care delivery, particularly in settings with limited specialist availability and developing stroke systems. Further studies using more robust analytical and prospective designs are needed to better define these associations.

## Figures and Tables

**Table 1:** Baseline Demographic and Clinical Characteristics of Stroke Patients.

Variables	Cases Mean ± SD, N (%)
Number of cases	573
Age	68.79 ± 13.77
Sex	
female	262 (45.7%)
male	311 (54.3%)
Stroke Workflow Period	
Neurohospitalist Period (1. period)	284 (49.6%)
Stroke Practitioners Period (2. period)	289 (50.4%)
Application Year	
2020	103 (18.0%)
2021	88 (15.4%)
2022	127 (22.2%)
2023	109 (19.0%)
2024	146 (25.5%)
NIHSS	11.91 ± 6.45
Treatment Type	
IVT	348 (60.7%)
EVT	98 (17.1%)
IVT + EVT	127 (22.2%)
Day of admission	
Monday	73 (12.7%)
Tuesday	73 (12.7%)
Wednesday	71 (12.4%)
Thursday	57 (9.9%)
Friday	55 (9.6%)
Saturday	78 (13.6%)
Sunday	166 (29.0%)
Duty period (hours)	
00:00–08:00	110 (19.3%)
08:00–16:00	230 (40.4%)
16:00–24:00	229 (40.2%)
DNT (minutes)	46.46 ± 27.70
DNT, median (IQR)	41 (30–57)
DNT category	
<60 min	325 (75.8%)
>60 min	104 (24.2%)
DPT (minutes)	114.24 ± 98.12
DPT, median (IQR)	105 (75–130)
ICU Transfer	
no	442 (77.1%)
yes	131 (22.9%)
LVO	
no	148 (26.1%)
yes	420 (73.9%)
3-month mRS Score	
0	117 (23.2%)
1	33 (6.5%)
2	64 (12.7%)
3	56 (11.1%)
4	66 (13.1%)
5	23 (4.6%)
6	146 (28.9%)
3-month mRS Score Group	
no-disability (mRS 0–2)	214 (42.4%)
disability-present (mRS 3–5)	145 (28.7%)
death (mRS 6)	146 (28.9%)

NIHSS, National Institutes of Health Stroke Scale; IVT, intravenous thrombolysis; EVT, endovascular thrombectomy; DNT, door-to-needle time; DPT, door-to-puncture time; ICU, intensive care unit; LVO, large vessel occlusion; mRS, modified Rankin Scale. Data are presented as mean ± standard deviation (SD) or number (percentage), as appropriate. The number of observations may vary across variables because analyses were performed using an available-case approach and missing data were not imputed.

**Table 2:** Clinical Characteristics and Outcomes between Neurohospitalist-Led and Stroke Practitioner-Led Periods.

	Neurohospitalist Mean ± SD or (%)	Stroke Practitioner Mean ± SD or (%)	*p*-Value
Number of cases	284 (49.6%)	289 (50.4%)	
Age (years)	66.82 ± 14.20	70.73 ± 13.06	0.002
Sex			0.287
female	126 (44.4%)	136 (47.1%)	
male	158 (55.6%)	153 (52.9%)	
NIHSS	12.09 ± 6.35	11.72 ± 6.55	0.486
Last Known Well (minutes)	292.79 ± 236.22	217.71 ± 250.07	<0.001
Wake-up stroke			0.029
no	196 (98.5%)	259 (94.9%)	
yes	3 (1.5%)	14 (5.1%)	
Treatment type			<0.001
IVT	136 (48.0%)	212 (73.4%)	
EVT	77 (27.1%)	21 (7.3%)	
IVT + EVT	71 (25.0%)	56 (19.4%)	
Day of admission			<0.001
Monday	26 (9.2%)	47 (16.3%)	
Tuesday	25 (8.8%)	48 (16.6%)	
Wednesday	23 (8.1%)	48 (16.6%)	
Thursday	29 (10.2%)	28 (9.7%)	
Friday	24 (8.5%)	31 (10.7%)	
Saturday	30 (10.6%)	48 (16.6%)	
Sunday	127 (44.7%)	39 (13.5%)	
Duty period (hours)			0.005
00:00–08:00	67 (23.9%)	43 (14.9%)	
08:00–16:00	97 (34.6%)	133 (46.0%)	
16:00–24:00	116 (41.4%)	113 (39.1%)	
DNT (minutes)	46.01 ± 24.18	46.76 ± 29.76	0.952
DNT category			0.124
<60 min	121 (72.5%)	204 (77.9%)	
>60 min	46 (27.5%)	58 (22.1%)	
DPT (minutes)	108.22 ± 45.71	124.36 ± 149.46	0.704
LVO			0.108
no	66 (23.6%)	82 (28.5%)	
yes	214 (76.4%)	206 (71.5%)	

NIHSS, National Institutes of Health Stroke Scale; IVT, intravenous thrombolysis; EVT, endovascular thrombectomy; DNT, door-to-needle time; DPT, door-to-puncture time; LVO, large vessel occlusion. Data are presented as mean ± standard deviation (SD) or number (percentage), as appropriate.

**Table 3:** Early and Late Outcomes between Neurohospitalist-Led and Stroke Practitioner-Led Periods.

	Neurohospitalist Mean ± SD or (%)	Stroke Practitioner Mean ± SD or (%)	*p*-Value
ICU Transfer			**0.002**
no	204 (71.8%)	238 (82.4%)	
yes	80 (28.2%)	51 (17.6%)	
24 h infarct expansion			0.451
no	167 (66.3%)	182 (67.2%)	
yes	85 (33.7%)	89 (32.8%)	
24 h hemorrhage			0.982
none	189 (74.7%)	202 (73.7%)	
hemorrhagic transformation	46 (18.2%)	51 (18.6%)	
parenchymal hematoma	16 (6.3%)	18 (6.6%)	
remote hemorrhage	2 (0.8%)	3 (1.1%)	
24 h remote infarct			0.746
no	280 (99.6%)	285 (99.7%)	
yes	1 (0.4%)	1 (0.3%)	
24 h brain edema			0.330
no	250 (92.9%)	231 (94.3%)	
yes	19 (7.1%)	14 (5.7%)	
24 h recanalization			0.087
occluded	44 (28.8%)	91 (35.8%)	
recanalized	109 (71.2%)	163 (64.2%)	
3-month mRS Score			0.208
0	64 (23.5%)	53 (22.7%)	
1	11 (4.0%)	22 (9.4%)	
2	38 (14.0%)	26 (11.2%)	
3	30 (11.0%)	26 (11.2%)	
4	40 (14.7%)	26 (11.2%)	
5	14 (5.1%)	9 (3.9%)	
6	75 (27.6%)	71 (30.5%)	
3-month mRS Category			0.490
no-disability (mRS 0–2)	113 (41.5%)	101 (43.3%)	
disability-present (mRS 3–5)	84 (30.9%)	61 (26.2%)	
death (mRS 6)	75 (27.6%)	71 (30.5%)	

ICU, intensive care unit; mRS, modified Rankin Scale. Data are presented as mean ± standard deviation (SD) or number (percentage). Statistically significant *p*-values (*p* < 0.05) are shown in bold. The number of observations may vary across variables because analyses were performed using an available-case approach and missing data were not imputed.

**Table 4:** Multivariable Logistic Regression Analysis of Variables Associated with Door-to-Needle Time < 60 min.

Variable	OR	95% CI	*p*-Value
Stroke Workflow Period	1.44	0.91–2.30	0.122
NIHSS	0.98	0.94–1.02	0.306
Age	0.99	0.97–1.01	0.231
Treatment (IVT vs. IVT + EVT)	0.93	0.69–1.23	0.598
LVO	0.69	0.40–1.18	0.179

DNT, door-to-needle time; OR, odds ratio; CI, confidence interval; NIHSS, National Institutes of Health Stroke Scale; LVO, large vessel occlusion; IVT, intravenous thrombolysis; EVT, endovascular thrombectomy. Multivariable logistic regression analysis was performed to identify variables independently associated with achievement of DNT < 60 min among patients receiving intravenous thrombolysis. Treatment type included IVT and IVT + EVT only, because DNT is defined exclusively in patients receiving intravenous thrombolysis. IVT served as the reference category. For Stroke Workflow Period, the neurohospitalist-led period served as the reference category. OR > 1 indicates a higher likelihood of achieving DNT < 60 min, whereas OR < 1 indicates a lower likelihood.

**Table 5:** Multivariable Logistic Regression Analysis of Variables Associated with Intensive Care Unit Transfer.

Variable	OR	95% CI	*p*-Value
Stroke Workflow Period	0.57	0.36–0.90	0.015
NIHSS	1.11	1.07–1.15	<0.001
Age	1.02	1.01–1.04	0.006
EVT only (vs. IVT)	1.89	1.04–3.44	0.037
IVT + EVT (vs. IVT)	1.16	0.67–2.03	0.593
LVO	1.58	0.85–2.91	0.145

ICU, intensive care unit; OR, odds ratio; CI, confidence interval; NIHSS, National Institutes of Health Stroke Scale; LVO, large vessel occlusion. Multivariable logistic regression analysis was performed to identify variables independently associated with ICU transfer. Treatment type was modeled using indicator variables, with IVT alone serving as the reference category. For Stroke Workflow Period, the neurohospitalist-led period served as the reference category. OR > 1 indicates a higher likelihood of ICU transfer, whereas OR < 1 indicates a lower likelihood. DNT category was not included in the final ICU transfer model because DNT is inherently undefined for EVT-only patients.

## Data Availability

The datasets generated and/or analysed during the current study are available in the Dataset Sharing Platform of Istinye University repository, [https://dataset.istinye.edu.tr/dataset?did=70, accessed on 18 May 2026].

## Supplementary Materials

The following supporting information can be downloaded at https://www.mdpi.com/article/10.3390/healthcare14131989/s1, Supplementary Table S1: Per-variable and per-period missingness for key study variables.

## Abbreviations

The following abbreviations are used in this manuscript:

AIS	Acute ischemic stroke
ED	Emergency department
CT	Computed tomography
CTA	Computed tomography angiography
IVT	Intravenous thrombolysis
EVT	Endovascular treatment
NIHSS	National Institutes of Health Stroke Scale
DNT	Door-to-needle time
DPT	Door-to-puncture time
ICU	Intensive care unit
mRS	Modified Rankin Scale
LVO	Large vessel occlusion
