# Supplementary material for: Real-World Comparison of Stroke Practitioner-Led and Neurohospitalist-Led Acute Ischemic Stroke Workflows
Source: Healthcare (Basel). 2026 Jul 3;14(13):1989. doi: 10.3390/healthcare14131989 (PMC13362298; doi:10.3390/healthcare14131989)
Supplement: Supplementary file 1 [file healthcare-14-01989-s001.zip › healthcare-4355890-supplementary.pdf]

**Supplementary Table S1.** Per-variable and per-period missingness for key study variables.

| Variable | Neurohospitalist available n/N (%) | Neurohospitalist missing n/N (%) | Stroke practitioner available n/N (%) | Stroke practitioner missing n/N (%) |
|---|---|---|---|---|
| Application Year | 284/284 (100.0%) | 0/284 (0.0%) | 289/289 (100.0%) | 0/289 (0.0%) |
| Gender | 284/284 (100.0%) | 0/284 (0.0%) | 289/289 (100.0%) | 0/289 (0.0%) |
| NIHSS | 282/284 (99.3%) | 2/284 (0.7%) | 282/289 (97.6%) | 7/289 (2.4%) |
| Age | 284/284 (100.0%) | 0/284 (0.0%) | 289/289 (100.0%) | 0/289 (0.0%) |
| Door-to-needle time | 167/284 (58.8%) | 117/284 (41.2%) | 262/289 (90.7%) | 27/289 (9.3%) |
| Door-to-puncture time | 120/284 (42.3%) | 164/284 (57.7%) | 72/289 (24.9%) | 217/289 (75.1%) |
| Last Known Well | 283/284 (99.6%) | 1/284 (0.4%) | 277/289 (95.8%) | 12/289 (4.2%) |
| Treatment type | 284/284 (100.0%) | 0/284 (0.0%) | 289/289 (100.0%) | 0/289 (0.0%) |
| ICU transfer | 284/284 (100.0%) | 0/284 (0.0%) | 289/289 (100.0%) | 0/289 (0.0%) |
| Wake-up stroke | 199/284 (70.1%) | 85/284 (29.9%) | 273/289 (94.5%) | 16/289 (5.5%) |
| LVO | 280/284 (98.6%) | 4/284 (1.4%) | 288/289 (99.7%) | 1/289 (0.3%) |
| 24-hour infarct expansion | 252/284 (88.7%) | 32/284 (11.3%) | 271/289 (93.8%) | 18/289 (6.2%) |
| 24-hour hemorrhage | 253/284 (89.1%) | 31/284 (10.9%) | 274/289 (94.8%) | 15/289 (5.2%) |
| 24-hour remote infarct | 281/284 (98.9%) | 3/284 (1.1%) | 286/289 (99.0%) | 3/289 (1.0%) |
| 24-hour brain edema | 245/284 (86.3%) | 39/284 (13.7%) | 269/289 (93.1%) | 20/289 (6.9%) |
| 24-hour recanalization | 153/284 (53.9%) | 131/284 (46.1%) | 254/289 (87.9%) | 35/289 (12.1%) |
| 3-month mRS | 272/284 (95.8%) | 12/284 (4.2%) | 233/289 (80.6%) | 56/289 (19.4%) |
| 3-month mRS category | 272/284 (95.8%) | 12/284 (4.2%) | 233/289 (80.6%) | 56/289 (19.4%) |

Abbreviations: ICU, intensive care unit; LVO, large vessel occlusion; mRS, modified Rankin Scale. Percentages were calculated using the total number of patients within each workflow period as the denominator. Missing data were not imputed, and analyses were conducted using an available-case approach.
